# Genus-targeted markers for the taxonomic identification and monitoring of coagulase-positive and coagulase-negative *Staphylococcus* species

**DOI:** 10.1007/s11274-024-04121-9

**Published:** 2024-10-03

**Authors:** S. Jiménez-Velásquez, M. E. Pacheco-Montealegre, L. Torres -Higuera, L. Uribe-Gutiérrez, D. Burbano-David, L. L. Dávila-Mora, C. Renjifo-Ibáñez, A. Caro-Quintero

**Affiliations:** 1 Livestock Microbiology Laboratory, Tibaitatá Research Center, Corporación Colombiana de Investigación Agropecuaria (AGROSAVIA), Km 14 vía Bogotá a Mosquera, Cundinamarca, Colombia; 2 Departamento de Biología, Facultad de Ciencias, Max Planck Tandem Group in Holobionts, Universidad Nacional de Colombia, Bogotá, Colombia

**Keywords:** Epidemiology, Molecular typing, Phylogenetics, Protein-coding genes, Staphylococcus

## Abstract

*The Staphylococcus* genus comprises multiple pathogenic and opportunistic species that represent a risk to public health. Epidemiological studies require accurate taxonomic classification of isolates with enough resolution to distinguish clonal complexes. Unfortunately, 16 S rRNA molecular analysis and phenotypic characterization cannot distinguish all species and do not offer enough resolution to assess intraspecific diversity. Other approaches, such as Multilocus Sequence Tagging, provide higher resolution; however, they have been developed for *Staphylococcus aureus* and a few other species. Here, we developed a set of genus-targeted primers using five orthologous genes (*pta*,* tuf*,* tpi*,* groEs*, and *sarA*) to identify all *Staphylococcus* species within the genus. The primers were initially evaluated using 20 strains from the Collection of Microorganisms of Interest in Animal Health from AGROSAVIA (CMISA), and their amplified sequences were compared to a set of 33 *Staphylococcus* species. This allowed the taxonomic identification of the strains even on close species and the establishment of intraspecies diversity. To enhance the scope and cost-effectiveness of the proposed strategy, we customized the primer sets for an Illumina paired-end amplicon protocol, enabling gene multiplexing. We assessed five genes across 177 strains, generating 880 paired-end libraries from the CMISA. This approach significantly reduced sequencing costs, as all libraries can be efficiently sequenced in a single MiSeq run at a fraction (one-fourth or less) of the cost associated with Sanger sequencing. In summary, this method can be used for precise identification and diversity analysis of *Staphylococcus* species, offering an advancement over traditional techniques in both resolution and cost-effectiveness.

**Graphical Abstract:**

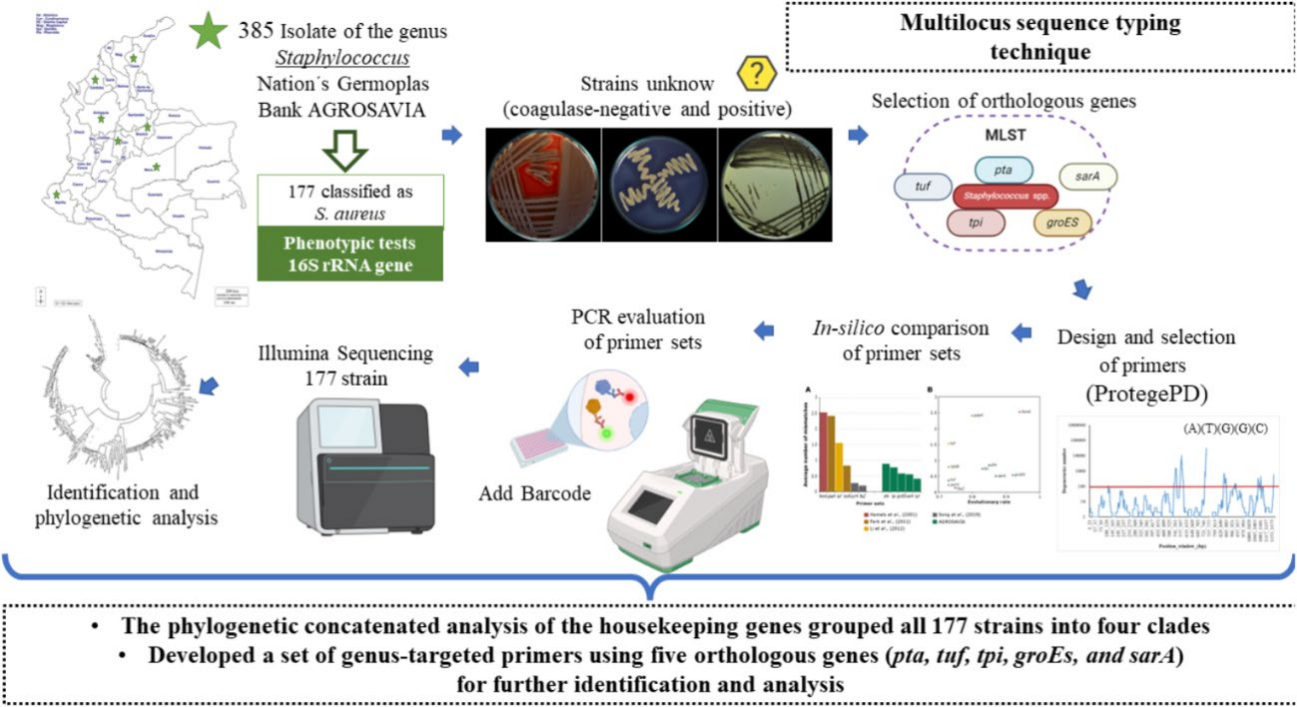

**Supplementary Information:**

The online version contains supplementary material available at 10.1007/s11274-024-04121-9.

## Introduction

*Staphylococcus* is a genus of Gram-positive bacteria that usually inhabit the skin and mucosa in humans and animals (Becker et al. 2014). Some species may behave as opportunistic pathogens due to their ability to express virulence factors, causing important infectious diseases in humans, livestock, and domestic animals (Gómez-Sanz et al. 2019; Nowakiewicz et al. 2016). These bacteria are usually classified based on the ability of the coagulase enzyme to coagulate blood plasma in vitro (Mehmeti et al. 2016; Pumipuntu et al. 2017). Among the coagulase-positive (CoPS) group, there are several species, such as *S. aureus*,* S. delphini*,* S. intermedius*,* S. lutrae*,* S. pseudintermedius*,* S. coagulans*, and two coagulase-variable, *S. hyicus* and *S. agnetis* (https://www.bacterio.net/). On the other hand, coagulase-negative (CoNS) species are a heterogeneous group with more than 40 species known to date (Asante et al. 2020; Madhaiyan et al. 2020; Nunes et al. 2016; Wanecka et al. 2018). The phenotypic variability in isolates belonging to the same species or in genetically closely related species, such as morphology, growth characteristics, ability to metabolize substrates, and antimicrobial resistance, has historically made their classification challenging either by manual or automated phenotypic methods (Adkins et al. 2017; Vanderhaeghen, Piepers, Leroy, Coillie, et al. 2015; Zadoks and Watts 2009).

In recent years, methods based on protein profiling, such as matrix-assisted laser desorption ionization-time of flight mass spectrometry (MALDI-TOF MS), have been used to identify *Staphylococcus* for both human (Matsuda et al. 2012) and animal isolates (Cameron et al. 2017; Tomazi et al. 2019). Although it is considered a straightforward, fast, and reliable methodology, it requires robust equipment, and its performance depends on a reference database (Wanecka et al. 2018). On the other hand, genotypic identification such as 1*6S rDNA* gene sequencing (Takahashi et al. 1999) shows limited resolution in distinguishing closely related species and, therefore, establishing evolutionary relationships (Adkins et al. 2017; Lamers et al. 2012).

On the other hand, restriction fragment polymorphism analysis (PCR-RPFL) of *gap* (Park et al. 2011; Srednik et al. 2015) and *groEL* (Raspanti et al. 2016) genes has been used to characterize isolates of animal origin. Other genotypic fingerprinting methods used up to date include intergenic transfer RNA spacer PCR (tDNA-PCR), 16–23S rDNA gene internal transcribed spacer PCR (ITS-PCR), and palindromic element PCR (rep-PCR) (Krishnamoorthy et al. 2016). Also, sequencing of protein-coding genes has been used for identification. Among these, the superoxide dismutase A (*sodA*) (Abdul-Aziz et al. 2015), *dnaJ* (Shah et al. 2007), rpoB (Drancourt and Raoult 2002), and *tuf* genes (Hwang et al. 2011).

In recent years, with sequencing platforms and the development of more cost-effective tools, whole genome sequencing (WGS) has become an ideal tool for microbiological identification (Vanderhaeghen, Piepers, Leroy, Van Coillie, et al. 2015). In agreement, recent studies have used WGS to establish phylogenetic relationships between *Staphylococcus* species(Naushad et al. 2016) as well as for CoNS genotyping (Naushad et al. 2019). However, Latin American countries are expected to use these technologies only in elite strains, which aim to highlight important biological attributes prior to the identification of isolates by less expensive methodologies. Here, we present the design and validation of primer sets for the amplification and sequencing of housekeeping genes. These primer sets can successfully amplify target species of clinical significance in human and animal health, within the CoPS and CoNS groups. These primers allowed diversity differentiation within the genus and can potentially be used in a novel MLST scheme to analyze several *Staphylococcus* species simultaneously. Furthermore, these primers can be easily adapted for high-throughput sequencing and used to assess genus diversity. Using the primers and strategies proposed here will contribute to identifying and understanding the epidemiological dynamics of different *Staphylococcus* species in livestock and human studies.

## Materials and methods

### Isolates

The strains used in this study belong to the collection of Microorganisms of interest in Animal Health-CMISA from the Nation’s Germplasm Bank System for Food and Agriculture, from the Colombian Corporation for Agricultural Research - Agrosavia. The collection has 385 strains of the genus *Staphylococcus*, obtained from dual-purpose systems and specialized dairies in Antioquia, Cundinamarca, Boyacá Cesar, Córdoba, Nariño, and Meta between the years 2002 and 2016. A total of 177 strains have been successfully classified as *S. aureus* by phenotypic tests (such as seeding in selective and differential media - Baird Parker, salty mannitol and DNAase, catalase, and coagulase test) and identified up to species level by using an automated system VITEK 2 for Gram positives and amplification of a 791 bp region of the 16 S *rRNA* gene (Mason et al. 2001). However, the abovementioned methods have not accurately identified the remaining 207 strains (coagulase-negative and positive). Accordingly, in this study, a group of 20 strains belonging to this group was selected initially for further identification and analysis (S1, supplementary file).

### Selection of genes

Available MLST schemes of different *Staphylococcus* species (Chassain et al. 2012; Enright et al. 2000; Song et al. 2019; Wang et al. 2003) were used to select housekeeping genes and orthologous genes present in the OrthoDB database (www.orthodb.org/) (S2, supplementary file ). For the inclusion of additional genes, the selection criteria were: (i) the presence of the gene in the *Staphylococcus* species listed in Supplementary file 3 (*Staphylococcus* species genomes used in the study). (ii) a slow rate of evolution, and (iii) its presence as a single copy gene. For the latter, the copy number was confirmed through BLASTn (Altschul et al. 1990) against available *Staphylococcus* genomes at NCBI (S3, supplementary file). Homologs within the genomes had a sequence identity > 95% and an e-value ≤ 0.005.

To assess the discriminatory power of the method proposed, we checked the presence of all protein-coding gene (*pta*,* tpi*,* sarA*,* tufA*,* and groEs*) in 35 *Staphylococcus* genomes species through Blastp (Altschul et al. 1990) against available *Staphylococcus* genomes and *Mammaliicoccus* at NCBI (S5, supplementary file). We confirmed the presence of all the genes in a total of 65 genomes *Staphylococcus* species and in five of the genus *Mammaliicoccus*.

### Design and selection of primers

Primer design for the selected housekeeping genes was done based on previously reported work (Caro-Quintero and Ochman 2015). Briefly, the gene sequences were aligned by codons based on a percentage consensus threshold. It calculates the forward and reverse primers and their corresponding number of degeneracies for all the positions within the alignment. This methodology uses primers based on regions that maximize sequence conservation and flank polymorphic regions. This allows a broad amplification of the genes in the targeted taxonomic group and a higher taxonomic resolution. Only primers with less than 100 degeneracies were pre-selected to avoid non-specific amplification. Consequently, final primer sets were selected to amplify regions between 150 and 450 bp, making them suitable for high-throughput sequencing with Illumina MiSeq using 250 paired-end reads.

### In-silico comparison of primer sets

To assess the annealing specificity of the primers, an in-silico analysis was done between the selected primers and the targeted gene sequence (S3, supplementary file). The evaluated primers included the set designed in this study (AGROSAVIA set) and others previously reported for the *Staphylococcus* genus (Hamel et al. 2001; Li et al. 2012; Park et al. 2011; Song et al. 2019). Only primers that met the following criteria were analyzed: less than five mismatches with the alignment and no mismatches at the 3’ end of the primer. Mismatches per primer set were quantified, especially in cases where one of the primers did not seem to anneal correctly to the target sequence. The average number of mismatches per set was quantified for the primer sets that successfully annealed to the target sequences. This work was done using Geneious Prime 2019.1.3 (https://www.geneious.com), with a modified version of Primer3 2.3.7 (Untergasser et al. 2012). Values of the evolutionary rate of orthologs were obtained for each set of ortholog genes from the OrthoDB database (www.orthodb.org/).

### PCR evaluation of primer sets

The experimental evaluation of the synthesized primers was carried out by PCR amplification of the 20 selected strains (S1, supplementary file), with ATCC 25,923 *Staphylococcus aureus* as the reference strain. DNA from a single bacterial colony was extracted from a culture on BHI agar using the commercial PureLink^®^ Genomic DNA kit for Gram-positive (Invitrogen) and UltraClean^®^ Blood DNA Isolation Kit (Non-Spin) from MoBio, according to the manufacturer’s instructions.

PCRs were performed in a C1000 BioRad^®^ thermocycler in 50 ul volume, containing 2 ul of genomic DNA normalized to a concentration of 30 ng/ul, 1X of 10X Buffer, 0.2 mM dNTPs, 4mM MgCl_2_,10 pml of each primer and 1 U of Taq DNA polymerase, recombinant (INVITROGEN, USA). The amplification cycles were at 95 °C for initial denaturation for 5 min, followed by 30 cycles at 95 °C for 1 min, annealing was done at 55 °C for 1 min, extension at 72 °C for 1 min and final extension at 72 °C for 5 min. Amplification products were visualized by 1.5% agarose gel electrophoresis at 90 V for 50 min. For *the sarA* gene, the PCR mixed needed some modifications; in brief, for a 50 ul volume reaction, 1X of 10X Buffer, 0.25mM dNTPs, 5mM MgCl2, 10 pmol of each primer, and 1 U of Taq DNA polymerase, recombinant). Furthermore, for the target genes *groEs* and *sarA*, the number of PCR cycles was adjusted (30 to 35 cycles) to increase the amplified strains successfully. Finally, all the PCR products were sequenced by primer extension using Sanger technology in both directions.

### Identification and phylogenetic analysis of staphylococcal strains subset

Sequences were processed using Geneious Prime v2019.1.3 sequence editor (https://www.geneious.com). Consensus sequences obtained from selected genes were confirmed to correspond functionally and taxonomically to the genetic targets in *Staphylococcus* sp. using BLASTx and MEGABLAST (https://blast.ncbi.nlm.nih.gov/Blast.cgi). Sequences of each genetic target were aligned with reference genes extracted from the representative genomes (S3, supplementary file), using MUSCLE (Edgar 2004) All alignments were concatenated using the Sequence Matrix v1.8 program. Phylogenetic reconstruction was performed using the Maximum Likelihood method based on the Kimura 2-parameter model, and the Bootstrap method was calculated with 1,000 iterations using the MEGA7 program (Kumar et al. 2016). Evolutionary divergence for the concatenated genes was calculated as the number of base substitutions per site (P-distance) between the obtained sequences and the references; these distances were represented as a heatmap using the ‘pheatmap’ package of R- 3.6.1.

### Evaluation of a larger collection of coagulase-negative and positive staphylococci strains using primer sets adapted to Illumina

After the initial standardization and primer selections, the sets of primers were adapted for high-throughput sequencing with Illumina MiSeq. The primer pairs that produced the most reliable results were synthesized with adaptor sequences attached to their 5′-ends. These adaptor sequences include a phase and a linker region, enabling primer sets to create amplicons apt for barcoding and simultaneous analysis of multiple samples (Faith et al. 2013). The library preparation was done for 177 coagulase-negative and positive staphylococci strains from the CMISA collection as follows. For DNA extraction of the strains, DNeasy^®^Power Soil Kit (Qiagen) was used according to the manufacturer´s instructions. The quality and integrity of DNAs obtained were evaluated through electrophoresis gel, and their concentration was measured using a NanoDropTM 1000 Spectrophotometer (Thermo Fisher Scientific, DE, USA). DNA concentration was adjusted to 30 ng/µl.

For library preparation, a two-step PCR procedure was followed. In the first PCR, the selected primers set were modified by including a linker region of 100 pb in the 5´ end. PCR reactions were conducted with Taq DNA Polymerase (Invitrogen™, Carlsbad, CA, USA) in 25 µl reaction, mixtures containing 1U of Taq Polymerase, 1X of 10X buffer (Invitrogen™, Carlsbad, CA, USA), 0.2 µM of each primer, 0.2 mM of dNTP mix, four mM MgCl_2_, and 30 ng of template DNA. All the primers were used at a final concentration of 0.25 mM/µl in a 25 µL reaction volume, and amplification conditions were the same as described above. All PCR products were purified following the protocol of Agentcourt^®^ AMPure^®^ XP. The second PCR added barcodes containing unique sequences for tagging each amplicon and the Illumina i5 and i7 capture sequences. This PCR was carried out by adding five µl of the previous amplicon, 1 µl (10 µM) of each of the forward and reverse barcode primers, and was performed using the same conditions described above for the first PCR, setting the number of cycles to 12. After visualization of PCR products in electrophoresis gel, these were purified following the protocol of Agentcourt^®^ AMPure^®^ XP and quantified on a NanoDropTM 1000 Spectrophotometer (Thermo Fisher Scientific, DE, USA). The purified amplicons were pooled, adjusted to the same concentration, and pair-end sequenced (250 nt PE reads) on the Illumina MiSeq System using a commercial service (Macrogen, Seoul, South Korea).

### Processing of reads

Quality control of amplicon sequencing reads was performed using FastQC v. 0.11.2. Primers and low-quality nucleotides from sequences were removed using Trimmomatic v 0.36. Demultiplexing, merging paired-end reads, and OTU clustering were performed using Qiime2 v2019-7.

### Data analyses

After an analysis of sequences, the most abundant sequence per library was used to represent the corresponding gene and strain. All the selected sequences were compared to sequences present in the database using the NCBI non-redundant nucleotide database nt, employing Blast (https://blast.ncbi.nlm.nih.gov/Blast.cgi), and the identity of the closest match was obtained. Sequences of each genetic target were aligned with reference genes extracted from the representative genomes (S3, supplementary file) using Clustal W. Phylogenetic reconstruction was performed by gene and also concatenated using the Maximum Likelihood method based on the Kimura 2-parameter model, and the Bootstrap method was calculated with 1,000 iterations using MEGA X program (Kumar et al. 2016).

## Results

### Molecular markers for the identification of *Staphylococcus* species

A set of eight single-copy genes were selected for primer design. Conserved and polymorphic regions were identified for each group, and forward and reverse primers were designed (Table 1). Only primers with less than 100 degeneracies were pre-selected to avoid non-specific amplification. An in-silico alignment of the primers against the targeted genes of the 33 *Staphylococcus* genomes showed few mismatches. Comparison between the alignment of the AGROSAVIA set and those previously reported (Hamel et al. 2001; Li et al. 2012; Park et al. 2011; Song et al. 2019) showed that our sets had a lower mismatch count.

**Table 1:** Primer sets designed for identification of *Staphylococcus* sp

Gen	Name	Primer Sequences (5’ --> 3’)	GC (%)	Amplicon Size (bp)
*groES*	groes2F*	AGAACAAACAACDAAAAGYGG	37.3	172
	groes4R**	TTCDGTDCCNGCATATTGTTG	43.7	
*pta*	pta1F	GGNAAAGCNACWGAAGAACAA	42.9	355
	pta4R	CDGAACCNTTHGTWGADAAGC	45.2	
*sarA*	sara2F	YGARGARTTYGCDGTDTTAAC	41.3	169
	sara4R	WCKTTCATCNTDTTCRTTACG	37.3	
*tpi*	tpi2F	GTGCNCCWACNATTCAATTAG	42.9	417
	tpi4R	ATTTACCWGTNCCRATHGCCC	49.2	
*tuf*	tuf2F	TAAACAAAGTTGACATGGTWG	33.3	400
	tuf5R	TAGTCTAATAAYTTACGGAAC	31.0	
*gmK*	gmk1F	ATAYGCNGARTATGTNGG	33,33	336
	gmk5R	CDGCNCGTGAYTTAGGNC	33,33	
*pyrR*	pyrR1F	CNCATGAAATHTTDGART	33,33	387
	pyrR4R	TNCCNACAAARTCTGCDC	38,89	
*glpF*	glpf4F	CACGNGCHGGRTTAATNG	44,44	241
	glpf2R	AYGTAYTTACCDCAHTGG	55,56	

F*: forward; R**: reverse

**Fig. 1:**
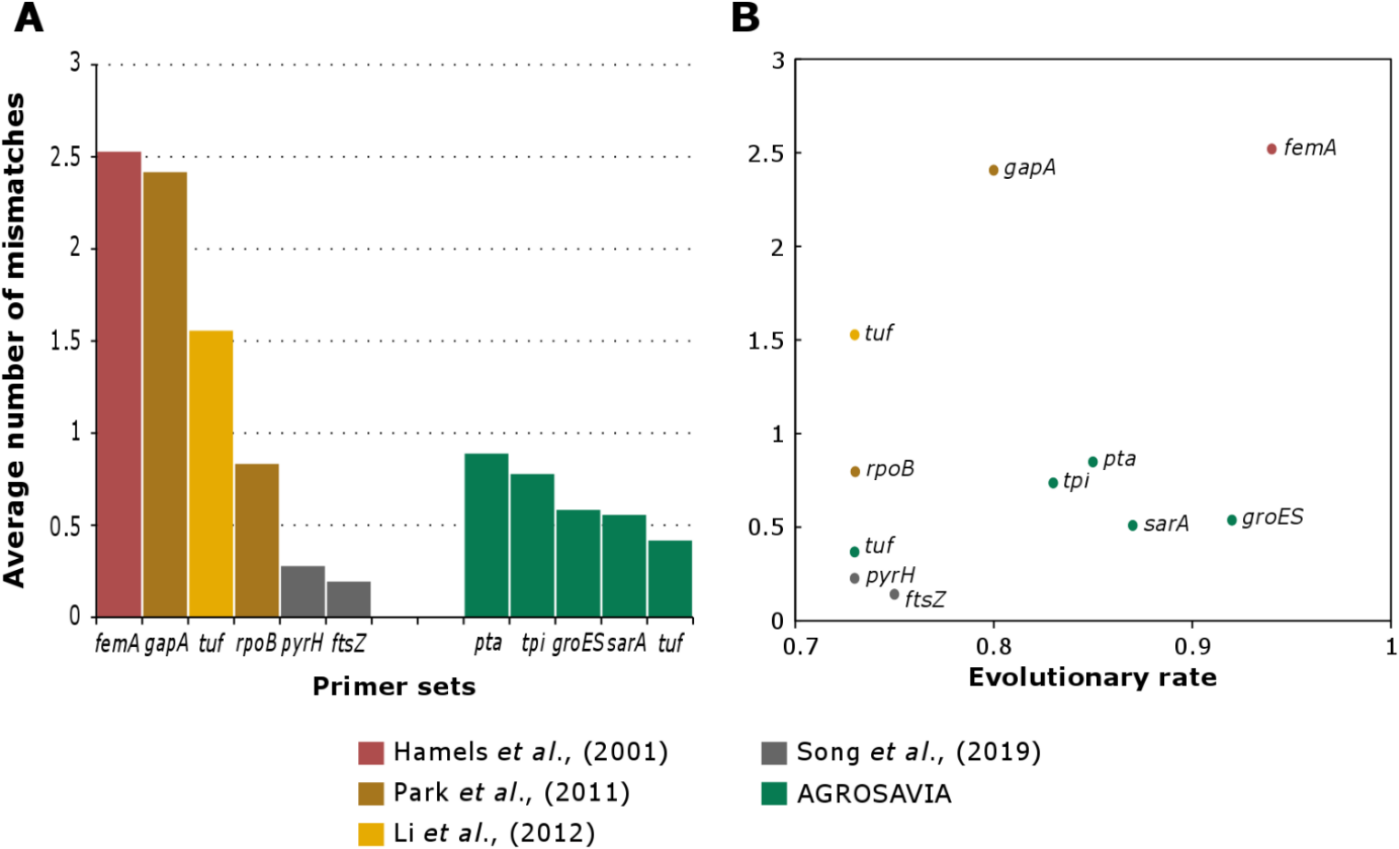
Average number of mismatches between the primer sets and the orthologs from the 33 evaluated genomes (**Panel A**). Assess the relationship between the evolutionary rate and the number of mismatches (**Panel B**)

**Fig. 2:**
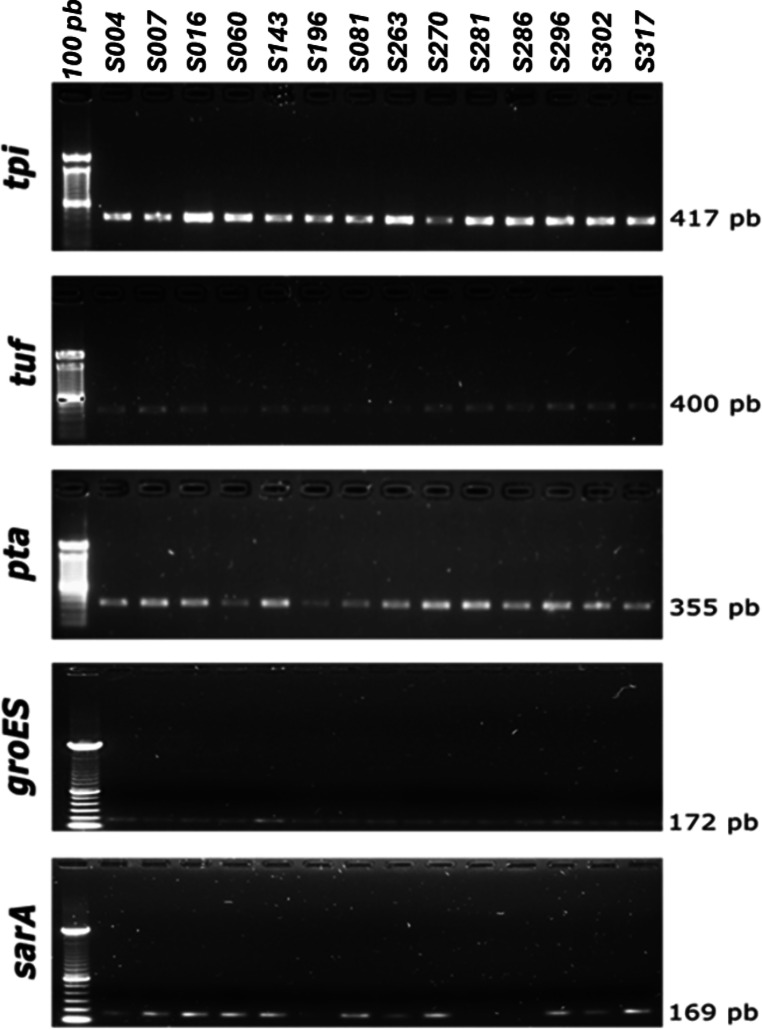
Amplification of the *tpi*,* tuf*,* pta*,* groEs*,* and sarA* genes in *Staphylococcus* strains selected to evaluate the genetic targets

The average mismatches of the primers presented in this study ranged from 0.41 to 0.89 for each first pair, in contrast to values ranging from 0.19 to 2.42 in the previously described set (Fig. 1A). A lower number of primer mismatches reduces the possibility of unsuccessful amplification of some groups. In general, the number of mismatches of the primer sets presented in this study was homogeneous despite their evolutionary rate (Fig. 1B). The PCR validation showed that five of the eight primer sets successfully amplified the expected DNA fragments (Fig. 2). The bands correspond to samples S196, S281 and S286, amplified using Illumina MiSeq technology with the *sarA* gene, can be seen in Supplementary file S6. The primers designed for the genes *pyrR*,* glpF*,* and gmk* showed unspecific amplification of DNA fragments in most strains tested; thus, they were discarded and not used for further analysis (data not shown).

### Identification and phylogenetic analysis of the staphylococcal strains subset

Identification of the strains was done by searching the sequences of the five genes against the nucleotide collection (nt) database using online BLAST (Altschul et al. 1990) (S4, supplementary file). Taxonomic identification was based on an identity higher than 95% with the query (Table 2). In most cases, the five genes agreed with the assignation. However, for some strains, multiple species matched our criteria. For instance, S004 was also classified as *S. devriesei*, yet only the sequence obtained from the *tuf*, pta and tpi gene showed > 99% similarity with the *S. devriesei* species. Likewise, S196 strains were classified as *S. xylosus*, but only the targets *pta* and *tpi* showed a similarity > 95%.

**Table 2:** Taxonomic identification of *Staphylococcus* species using the target genes. Taxonomic assignation based on the hits that showed identity > 95% in BLAST

Strain	Closest related genomes based on House-keeping genes
S004	*S. devriesei* strain CCUG 58,238 (MF678988.1)
S007	*S. haemolyticus* strain ATCC 29,970 (CP035291.1)
S016	*S. chromogenes* strain 20B (CP031471.1)
S060	*S. chromogenes* strain 20B (CP031471.1)
S081	*S. warneri* strain FDAARGOS (CP054017)
S196	*S. xylosus* strain 2 (CP031275.1)
S257	*S. epidermidis* strain ATCC 12,228 (CP043845.1)
S258	*S. epidermidis* strain ATCC 12,228 (CP043845.1)
S274	*S. saprophyticus* strain IUHSS04 (KM454817.1)
S281	*S. saprophyticus* strain 1 A (CP031196.1)
S286	*S. agnetis* strain 12B (CP031266.1)
S296	*S. cohnii* strain FDAARGOS_334 (CP027422.1)
S302	*S. agnetis* strain 12B (CP031266.1)
S317	*S. agnetis* strain 12B (CP031266.1)
S318	*S. agnetis* strain 12B (CP031266.1)
S336	*S. chromogenes* strain 34B (CP031470.1)
S344	*S. agnetis* strain 12B (CP031266.1)
S143	*Mammaliicoccus sciuri*, strain FDAARGOS_285 (CP022046.2)
S263	*Mammaliicoccus sciuri*, strain FDAARGOS_285 (CP022046.2)
S270	*Mammaliicoccus sciuri* strain B9-58B (CP041879.1)

**Fig. 3:**
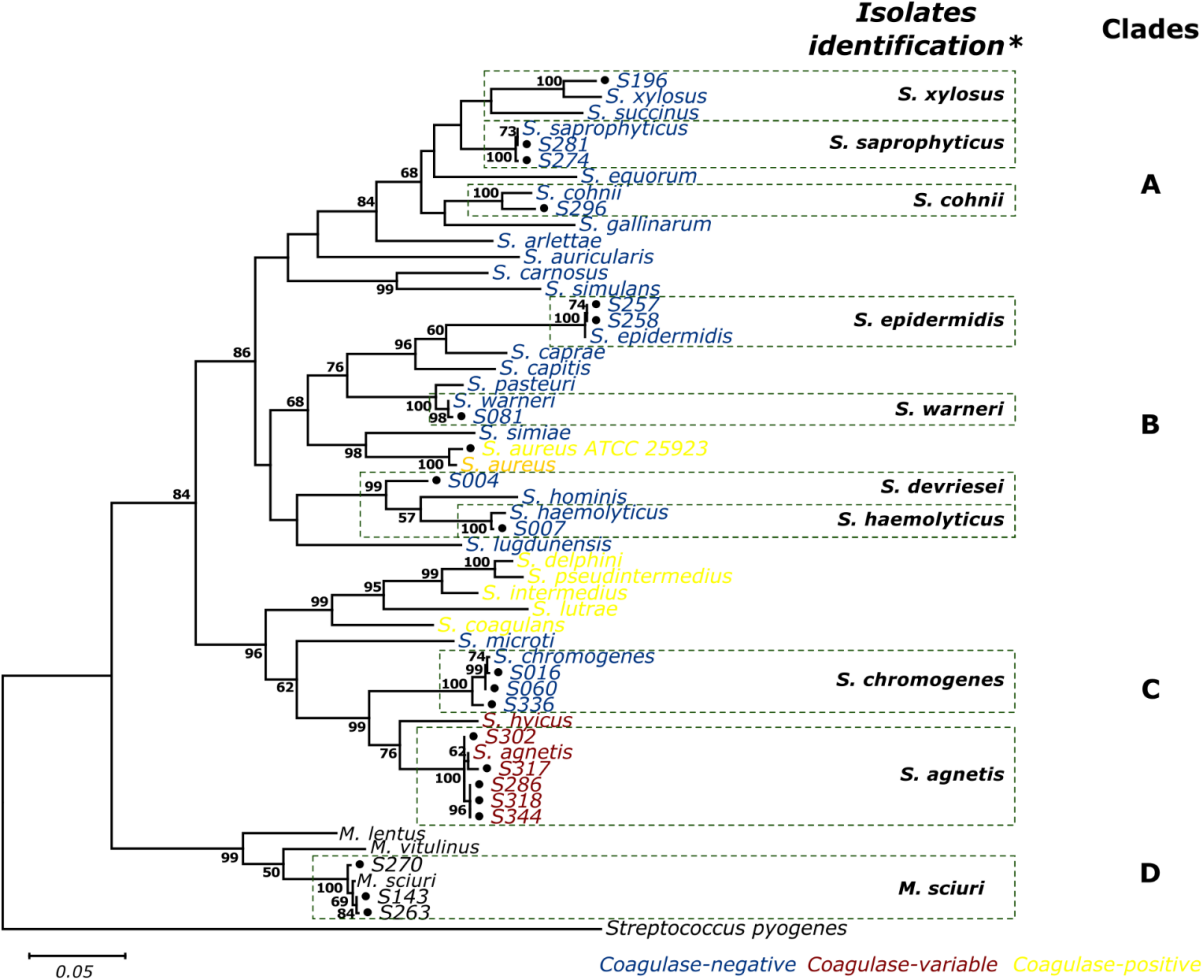
Molecular Phylogenetic analysis of 20 *Staphylococcus* strains using concatenated sequences of the five genes. The evolutionary history was inferred using the concatenated genes tuf, pta, groEs, sarA, and tpi sequences. The Maximum Likelihood method is based on the Kimura 2-parameter model. The percentage of trees, only > 50%, in which the associated taxa clustered together is shown next to the branches. All positions containing gaps and missing data were eliminated. There were a total of 1002 positions in the final dataset. *Streptococcus pyogenes* is the outgroup. In blue is the *Staphylococcus* coagulase-negative, in yellow is the coagulase-positive, and in red is the coagulase variable response

**Fig. 4:**
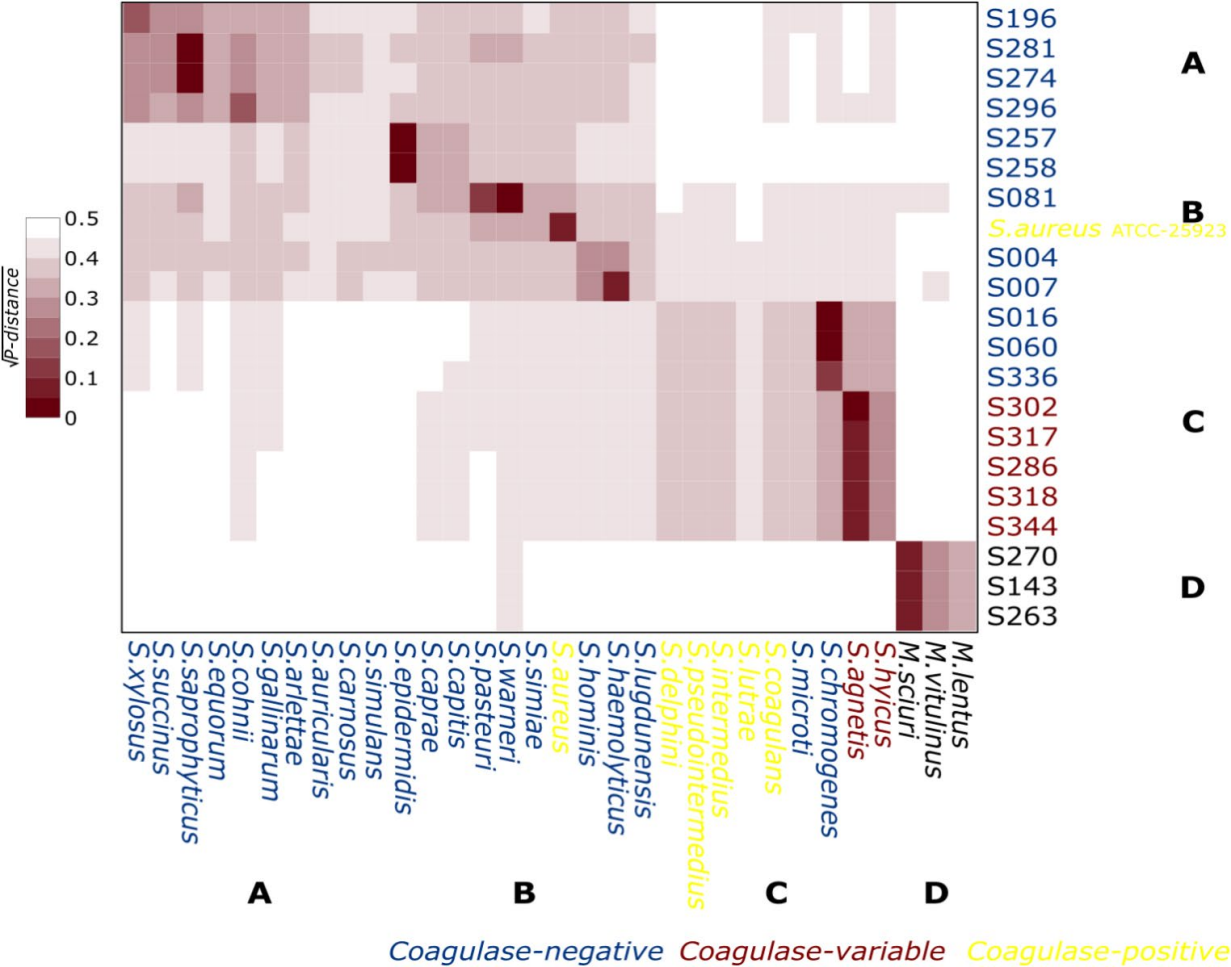
Estimates of evolutionary divergence between strains using concatenated gene sequences. The square root of the number of base substitutions per site (P-distance) between strains and reference species is shown. The analysis was conducted using the Kimura 2-parameter model. Evolutionary studies were conducted in MEGA7 (Kumar et al. 2016). In blue is the *Staphylococcus* coagulase-negative, in yellow is the coagulase-positive, and in red is the coagulase variable response

The phylogenetic concatenated analysis of the housekeeping genes *pta*,* tuf*,* tpi*,* groEs*, and *sarA* grouped the strains and the reference strains into four clades, named in this work as Clade A, Clade B, Clade C, and Clade D, which agree with the previously reported classification (Lamers et al. 2012; Naushad et al. 2016) (Fig. 3). Clade A is composed of S. *simulans*,* S. carnosus*,* S. auricularis*,* S. arlattae*,* S. gallinarum*,* S. cohnii*,* S. equorum*,* S. saprophyticus*,* S. succinus and S. xylosus*. The strain S196, identified as *S. xylosus*, S274, and S281, identified as *S. saprophyticus*, and S296, identified as *S. cohnii*, were included in this group. Clade B grouped the species S. *lugdunensis*, S. *haemolyticus*,* S. hominis*,* S. aureus*,* S. simiae*,* S. warneri*,* S. pasteuri*,* S. capitis*,* S. caprae*, and *S. epidermidis*. Strains identified as *Staphylococcus devriesei* (S004), *Staphylococcus haemolyticus* (S007), *Staphylococcus warneri* (S081), and *Staphylococcus epidermidis* (S257 and S258) were also grouped in Clade B. Clade C comprised coagulase positive species (*S. intermedius*,* S. delphini*,* S pseudintermedius*,* S. lutrae*,* and S. coagulans*), variable coagulase (*S. agnetis* and *S. hyicus*), and some coagulase-negative *Staphylococcus* strains (*S. chromogenes* and *S. microti*). Within this group were the strains S286, S302, S317, S318, and S344, which were identified as *S. agnetis*. Furthermore, we also found strains S016, S060, and S336 associated with *S. chromogenes* in this group. Clade D grouped *M. lentus*,* M. vitulinus*, and *M. sciuri* (S143, S263, and S270) with the strains identified as *S. sciuri*. Still, with the recent update of the taxonomy of the family *Staphylococcaceae*, these species were reassigned to the new genus *Mammaliicoccus* (Madhaiyan et al. 2020). For the taxonomic assignment, most strains presented a phylogenetic distance between 0 and 0.1 (Fig. 4, dark red color) to known references.

All sanger sequences were deposited in the GenBank database under accession numbers MT830621 to MT830639 for *groEs*, MT830640 to MT830657 for *pta*, MT830658 to MT830676 for *tpi*, MT830677 to MT830694 for tuf and MT880915 to MT880931 for *sarA*.

### Molecular characterization of an extensive strain collection using the gene marker scheme adapted to high-throughput sequencing

The molecular identification of genes adapted to high-throughput sequencing was applied to 177 strains. Strains were identified by searching the nucleotide collection (nt) database for the closest taxonomic match of the five targeted genes using online BLAST (Altschul et al. 1990). Best matches with an identity higher than 95% to the query were selected. In most cases, the taxonomic assignment of the five genes from the same strain agreed. However, for some strains, certain genes were assigned to a different species.

**Fig. 5:**
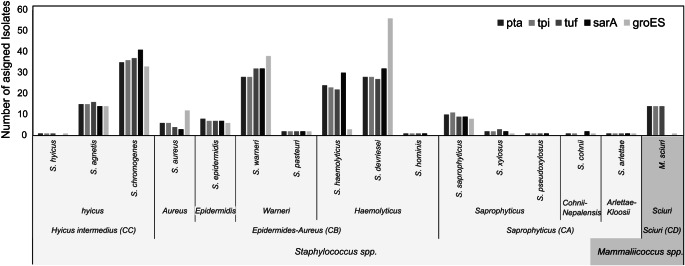
Phylogenetic separation of 177 strains with *pta*,* tuf*,* sarA*,* and groEs* gene in agreement with the proposed by Lammer. **A**. Clade (CA), **B**. clade (CB) and *Staphylococcus* species (spp.), **C**. clade (CC), **D**. clade (CD)

Utilizing this approach, the analysis of *pta*, *tpi*, and *tuf* genes revealed that most of the strains (163; 92%) were classified within the *Staphylococcus* genus, while 14 (8%) were categorized under the *Mammalicoccus* genus. Regarding the *groEs* gene, 176 strains (99%) were classified as *Staphylococcus* spp. with only 1 (1%) falling into the *Mammalicoccus* spp. category. In contrast, all 177 strains were classified as *Staphylococcus* spp. base on the *sarA* gene (Fig. 5).

The phylogenetic analysis allowed the grouping of isolates within each genus. In this study, isolate within the genus *Staphylococcus* were categorized into distinct clades designated as Clade A (Saprophyticus), Clade B (Epidermidis-Aureus) and Clade C (Hyicus-Intermedius). For strains classified within the genus *Mammalicoccus*, they were grouped into Clade D (Sciuri). Within the *Staphylococcus* clades, the isolates were further classified in clade-groups Saprophyticus, Cohnii-Nepalensis, Arlettae-Kloosii in Clade A, Aureus, Epidermidis, Warneri and Haemolyticus in Clade B, and Hyicus in Clade C. Meanwhile, within Clade D, all isolates were grouped within the Sciuri Clade-group (CD) (Fig. 5).

In clade A, the majority of the strains were classified as *S. saprophyticus*, ranging from 8 to 11 strains per gene. On average, two strains were classified as *S. xylosus*, and only one strain per gene was classified as *S. pseudoxylosus.* However, the strain (S196) was initially identified as *S. xylosus*. However, when evaluated using genetic markers adapted to high-throughput sequencing, it was reclassified as *S. pseudoxylosus* because the majority of genes exhibited > 95% similarity in Blast results, with the except of *groES.* Within the Cohnii-Nepalensis clade-group, one to two strains were classified as *S. cohnii*, except for the *tuf* gene. Similarly, one strain per gene belonged to the species *S. arlettae* within the Arlettae-Kloosii cluster group (Fig. 5).

In Clade B, six strains were classified into the Aureu*s* cluster group with *pta* and *tpi*, four with *tuf*, three with *sarA*, and a greater number with *groES*, totaling 12 strains. On average, 7 ± 1 strains belonging to the Epidermidis cluster group were classified as *S. epidermidis*. In the Warneri cluster group, most strains (> 28) were identified as *S. warneri*, while two were identified as *S. pasteuri* with each gene. For the Haemolyticus cluster group, on average, 23 ± 1 (13%) strains were identified as *S. haemolyticus* with the *pta*,* tpi* and tuf gene, 30 with *sarA* and tree with *groES*. Conversely, 56 strains were classified as *S. devriesei* with *groES* gene, and on average, 29 ± 2 were classified in this species with *pta*, *tpi*, *tuf* and *sarA*. Finally, for this clade-group, one strain was classified as *S. hominis* with the most genes, except with *groEs* as no strains belong to this species (Fig. 5).

In Clade C, the strains classified into the Hyicus cluster group, only one with *pta*, *tpi*, *tuf* and *groES* genes was classified as *S. hyicus*, and on average, 15 ± 1 strains were classified as *S. agnetis*, with most strains (> 33; 19%) identified as *S. chromogenes* with all the genes evaluated (Fig. 5). Finally, Clade D grouped the species into the *Mammalicoccus* genus, 14 (8%) was classified as *M. sciuri* with the *pta*, *tpi*, and *tuf* genes and only one with the *groES* gene (Fig. 5).

**Fig. 6:**
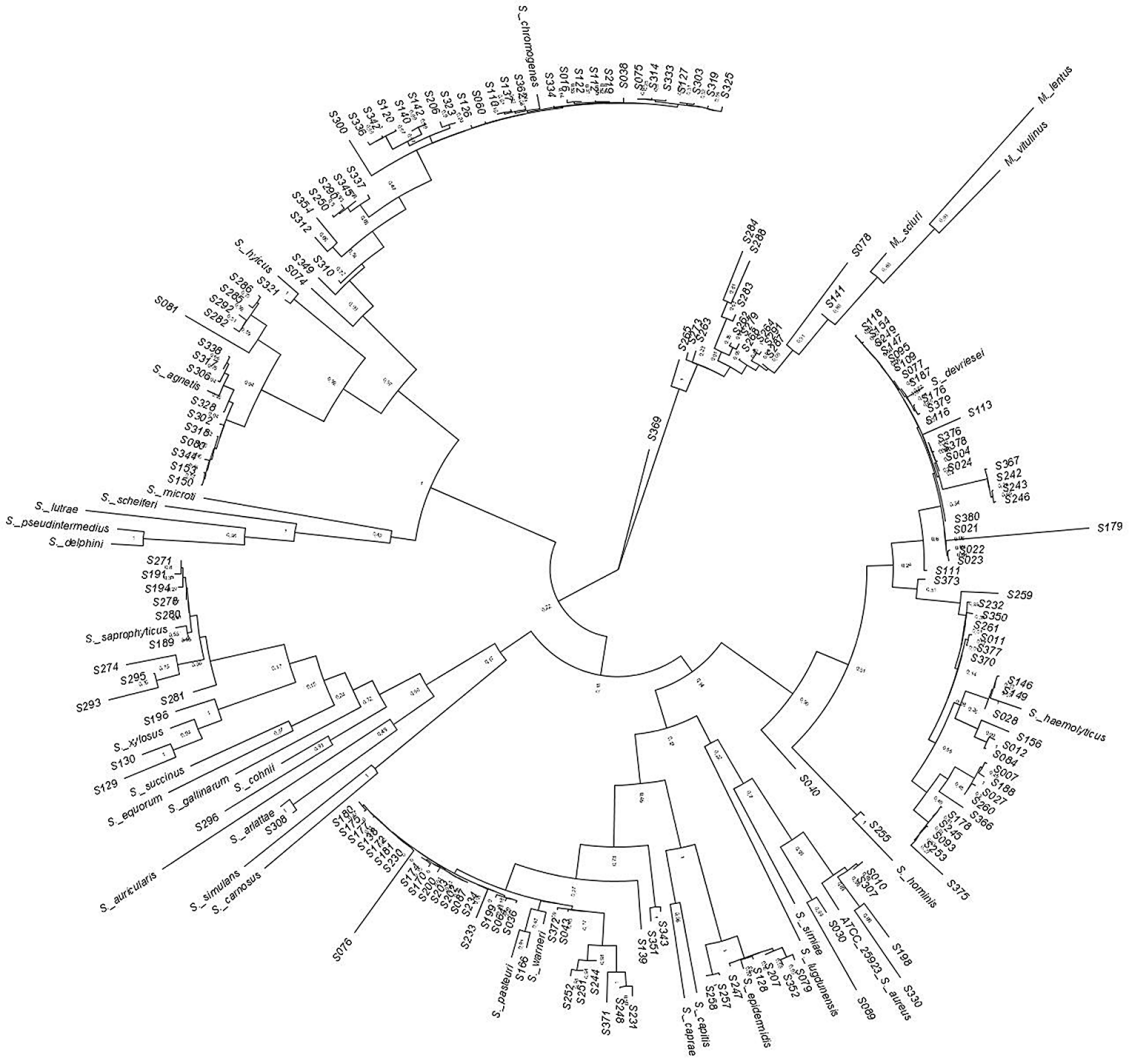
Molecular phylogenetic analysis of 177 isolates using concatenated sequences of the five genes *(tuf*,* pta*,* groEs*,* sarA and tpi).* The evolutionary history was inferred by using the Maximum Likelihood method and Kimura 2-parameter model. The tree with the highest log likelihood (-39108.15) is shown. Initial tree(s) for the heuristic search were obtained automatically by applying Neighbor-Join and BioNJ algorithms to a matrix of pairwise distances estimated using the Maximum Composite Likelihood (MCL) approach, and then selecting the topology with superior log likelihood value

In summary, the phylogenetic concatenated analysis of 177 strains from the CMISA collection facilitated their classification into 15 species within the genus *Staphylococcus* and one within the genus *Mammalicoccus*. Fifteen (8%) isolates were classified within clade CA, 97 (55%) within clade CB, 51 (29%) within clade CC, and 14 (8%) within clade CD. The majority of the strains were identified as *S. chromogenes* (35%), *S. warneri* (30%), *S. devriesei* (29%) and *S. haemolyticus* (23%) (Fig. 6). The strain S040 showed 100% similarity to *S. devriesei* based on the *tuf* and *sarA* gene, although other genes indicated different identification; however, it was classified as *S. devriesei* in the phylogenetic concatenated analysis. Strain S139 showed greater than > 95% similarity to *S. warneri*, while strains S343 and S351 were classified as *S. pasteuri* with 100% similarity according to the identification of each gene. These strains were categorized within the clade CB Epidermidis-Aureus. Finally, only the strain S369 showed different identifications with each gene, and therefore, in the concatenated analysis, it was excluded from the clade categorization.

All sequences have been deposited in the GenBank database under accession numbers OP747853 to OP748029 for *groEs*, OP958263 to OP958439 for *pta*, OP958440 to OP958616 for *tpi*, OP747676 to OP747852 for *tuf* and OP748030 to OP748206 for *sarA.*

## Discussion

Taxonomic identification of pathogenic and opportunistic microorganisms such as the genus *Staphylococcus* is known to represent a challenge, given the limitations of some phenotypic and molecular tools that are widely used (Adkins et al. 2017). In our previous studies, identification through an automated biochemical panel (VITEK2) and analysis of the 16 rRNA gene wasn’t enough to differentiate between the species *S. hyicus* and *S. agnetis*, or *furthermore*,* S. warneri*, which is commonly phenotypically classified as *S. chromogenes* with > 99% similarity to *S. pasteuri* (Hwang et al. 2011). These techniques probably show lower precision in identifying closely related positive and negative coagulase species of *Staphylococcus* (Nunes et al. 2016).

Primer design for identification and variant detection is challenging due to the difficulty of finding conserved regions in protein-coding genes within divergent groups. Here, we successfully adapted the previous work (Caro-Quintero and Ochman 2015) to design primers against Staphylococcus’s genes *pta*,* tuf*,* tpi*,* groEs*, and *sarA*. Our approach maximizes the recovery of sequence variants (amplified region), minimizing degeneracies within the designed primers. This advantage was shown by comparing primer mismatches of our set with another previously described for the *tuf* gene (Song et al. 2019), where our primers had three times less mismatches. Differences in mismatches can be associated with the nucleotide conservancy of the selected gene, where slow-evolving genes show a lower number of mismatches. This is the case of the previously reported primers for the genes *pyrH* and *ftsZ* (Song et al. 2019), which have a low number of mismatches but simultaneously have a slow evolutionary rate. However, the lower number of mismatches in our approach is not related to the higher conservancy of the genes used (lower evolutionary rate). A comparison of orthologs of both sets using OrthoDB (Kriventseva et al. 2019) shows that most target genes selected in our primer sets have a higher evolutionary rate than the ones proposed in previous works (Song et al. 2019).

The phylogenetic analysis of the concatenated sequences revealed four groups supported with a bootstrap between 86 and 100%, like those previously reported (Lamers et al. 2012). These large groups represent ancient events during the evolutionary history of *Staphylococcus* species, branching into smaller groups as evidenced by more recent events (Naushad et al. 2016). With the recent taxonomy update of the *Staphylococcaceae* family and the exclusion of some species to another genus, the structure found in the phylogeny of this work agrees with those previously reported (Madhaiyan et al. 2020).

In Clade A, the species *S. xylosus*,* S. saprophyticus*,* S. cohnii*,* S. gallinarum*,* S. succinus*,* S. equorum*, and *S. arlattae* agree to previously reported classifications (Ghebremedhin et al. 2008; Lamers et al. 2012). However, the species *S. auricularis* and *S. simulans* commonly form separate species groups (Becker et al. 2014). The Clade B, as expected, showed several subgroups, which is consistent with reported data by Naushad 2016, one of them among the species *S. epidermidis*, *S. caprae*, and *S. capitis*, probably because these three species share antigenic determinants associated with pathogenicity (Argemi et al. 2019). Another group comprising *S. pasteuri*, *S. warneri*, *S. hominis*, and *S. haemolyticus*, is similar to that previously reported (Becker et al. 2014; Lamers et al. 2012; Naushad et al. 2016) and differs from previous work by Shah et al. (2007), that report group an *S. haemolyticus* together with *S. hominis* as a separate group of species. The phylogenetic relationships between the species of *S. hominis*, *S. haemolyticus*, and *S. devriesei* could also be evidenced, facilitated by the inclusion of the strain S004, which was classified in this study as *S. devriesei*, in agreement with previous reports (Naushad et al. 2016; Schmidt et al. 2018). Another interesting subgroup was *S. simiae* and *S. aureus*, given that although these two species show a different reaction to coagulase, they are considered sister species (Becker et al. 2014; Suzuki et al. 2012). Finally, *S. lugdunensis* is considered a coagulase-negative species the closest to *S. aureus* in terms of pathogenicity (Argemi et al. 2019; Chassain et al. 2012). Clade C clusters the species *S. hyicus* and *S. agnetis*, which are considered phylogenetically close, although by phenotypic tests, they are often misclassified (Adkins et al. 2017). In the present study, it was possible to classify the more related strains (S286, S302, S317, and S344) to *S. agnetis* than to *S. hyicus*, proving the discriminatory capacity of the set of these genes to differentiate nearby species.

Some differences in the clustering topology were found compared to previous studies; these differences may result from the genetic targets used. As some of the genes might have engaged in homologous recombination with other species confounding the phylogenetic signal, previous studies have shown a different organization of *Staphylococcus* species that varies according to the gene selected for the study (Abdul-Aziz et al. 2015; Ghebremedhin et al. 2008; Shah et al. 2007).

Although constitutive genes have been used for species-specific identification and typing in *Staphylococcus* (Adkins et al. 2017), several molecular markers that could better reflect the genetic relationship between species is an improved strategy (Pérez-Losada et al. 2013). The molecular marker scheme based on protein-coding genes evaluated in the present study showed good discriminatory power for differentiating closely related species such as S. *capitis and S. caprae*,* S. pasteuri* and *S. warneri*,* S. saprophyticus*,* S. xylosus*. On the contrary, the identification obtained with the *sodA* gene exhibits low divergence within these species (Abdul-Aziz et al. 2015) and between variable coagulase species such as *S. agnetis* and *S. hyicus*, which – in turn- have shown similarity with the analysis of the rpoB gene > 99% in isolates of bovine origin (Adkins et al. 2017).

It is evident that multiple loci analysis techniques provide more information on organisms´ clonal and phylogenetic relationships (Lamers et al. 2012; Naushad et al. 2016). There are currently documented species-specific MLST schemes in *S. aureus* (Enright et al. 2000), *S. epidermidis* (Wang et al. 2003), *S. lugdunensis* (Chassain et al. 2012), *S. haemolyticus* (Cavanagh et al. 2012), *S. hominis* (Zhang et al. 2013), *S. pseudintermedius* (Solyman et al. 2013), *S. chromogenes* (Huebner et al. 2021) and recently *S. capitis* (Wang et al. 2022). To date, only one includes several *Staphylococcus* species (Song et al. 2019) but is directed to 18 different species. However, the target scheme we selected in this study could be used under the MLST approach, including the analysis of 33 different species of CoPS and CoNS. Considering the update of the taxonomy of the *Staphylococcaceae* family, the genetic target scheme presented in this study could become the first MLST scheme used to study populations of bovine origin of the genus *Mammaliicoccus* that would include the species *M. sciuri*,* M. lentus*, and *M. vitulinus*, different from previously proposed (Boonchuay et al. 2023; Schauer et al. 2021), that was developed for *M. sciuri* only.

In the present study, the primer sets selected were adapted for high-throughput sequencing with Illumina MiSeq for the taxonomic identification of the 177 coagulase-negative and positive staphylococci strains from the CMISA collection. The phylogenetic concatenated analysis from 177 strains agreed with that obtained previously with the 20 coagulase-negative and positive staphylococci strains. The taxonomic assignment of the five genes was consistent across most strains. However, for some strains, certain genes were assigned to a different species, this was especially evident for *sarA* and *groES* genes. When phylogenetic reconstruction was performed with each gene, inconsistent tree topology was observed, which might provide possible evidence of horizontal gene transfer (HGT) and recombination. This pattern was particularly evident between species of Hyicus-Intermedius and Epidermidis-Aureus groups, some species such as *S. devriesei* and *S. haemolyticus* seem to be the donors in the genetic exchange based on the higher number of assigned genes, which agree with previous reports (Smith and Andam 2021; Ikhimiukor et al. 2023). Furthermore, the patterns of HGT and recombination have been previously reported in at least 13 species of the CoNS group (Ikhimiukor et al. 2023); the species most frequently were *S. xylosus*,* S. chromogenes*,* S. hominis*, among others, similar to the present study. Genetic exchange is not considered random within and between species CoNS; on the contrary, these species have phage, plasmids, and mobilizable gene encoding of protein, which, through horizontal gene transfer and recombination, allow them to adapt a different environmental condition (Ikhimiukor et al. 2023). However, the use of several constitutive genes like molecular markers for species-specific identification, and the phylogenetic concatenated analysis can buffer the effect of recombination ADN in large numbers of pathogenic isolates (Hanage et al. 2005; Graña-Miraglia et al. 2018) similarly to the approach used in the present study.

Notably, the calculated costs of our approach reduce the cost of such molecular characterizations. In our case, sequencing 177 samples with five genetic targets might range between 4,400 and 8,800 dollars, depending on the local costs of Sanger sequencing. In contrast, the primer scheme adapted to high-throughput sequencing by Illumina NGS technology might be done for 1,500 dollars or lower.

The result showed that the cost was one-fourth of the price, which is appropriate for analyzing a large number of samples and less than 20 genetic targets (Rubio et al. 2020) (https://www.illumina.com/science/technology/next-generation-sequencing/ngs-vs-sanger-sequencing.html).

Another application for this primer scheme adapted to high-throughput sequencing is its use in the culture-independent studies of the diversity of these bacterial populations in natural environments or hosts (Edgar 2004). It is common to employ high-throughput sequencing with universal markers such as the 16 S rRNA gene (Taponen et al. 2019). However, using constitutive genes such as those evaluated in this study has shown to be critical in addressing species and intra-species (Taddei et al. 2021) diversity in human gut commensals and other hominoids (Caro-Quintero and Ochman 2015). It will provide a better understanding of these populations’ ecological and evolutionary dynamics over time (Moeller et al. 2016).

## Conclusions

The design of primers for amplification of orthologous genes that target broad taxonomic groups, such as species of the same genus, allowed us to establish with higher resolution the taxonomic affiliation of *Staphylococcus* isolates belonging to broad groups such as CoPS and CoNS to known species. At the same time, the obtained sequences have enough polymorphic sites to separate isolates into clonal groups. Adapting the five genes primer set to high-throughput sequencing with Illumina MiSeq allowed us to reduce the cost of molecular characterization to large collections by at least 75%. Analysis of this data enables the species identification of 177 isolates, providing clear separation even between closely related species, thanks to the discriminatory capacity of the selected genes.

In summary, the innovative primer design and adaptation to high-throughput sequencing presented here will significantly enhance the resolution of taxonomic classification in *Staphylococcus* isolates, enabling cost-effective species identification and clonal grouping with remarkable precision.

## Electronic supplementary material

Below is the link to the electronic supplementary material.

Supplementary Material 1

Supplementary Material 2

Supplementary Material 3

Supplementary Material 4

Supplementary Material 5

Supplementary Material 6

## Data Availability

No datasets were generated or analysed during the current study.
