# Supplementary material for: Genus-targeted markers for the taxonomic identification and monitoring of coagulase-positive and coagulase-negative Staphylococcus species
Source: World J Microbiol Biotechnol. 2024 Oct 3;40(11):333. doi: 10.1007/s11274-024-04121-9 (PMC11447098; doi:10.1007/s11274-024-04121-9)
Supplement: Supplementary file 1 — Supplementary Material 1 [file 11274_2024_4121_MOESM1_ESM.docx]

**S_1_**. Description of the *Staphylococcus* strains selected for the study.

| **Strains** | **Coagulase**  **test** | **Species determination by**  ***16S rRNA* (identity >99%)** | **Species determination by VITEK test % probability** |
| --- | --- | --- | --- |
| ***S004*** | - | *Staphylococcus devriesei (MF109902.1)* Staphylococcus haemolyticus (KY623167.1)* | *S. hominis*  *(93%)*** |
| ***S007*** | - | *Staphylococcus haemolyticus (MN512280.1) Staphylococcus hominis strain (MN252041.1)* | *S. haemolyticus (95%)* |
| ***S016*** | - | *Staphylococcus chromogenes (MN314469.1)* | *S. chromogenes (97%)* |
| ***S060*** | - | *Staphylococcus chromogenes (MT072170.1)* | *S. chromogenes (99%)* |
| ***S081*** | - | *Staphylococcus warneri (MK093025.1) Staphylococcus pasteuri (MN696510.1)* | *S. chromogenes (86%)* |
| ***S196*** | - | *Staphylococcus saprophyticus (MK743982.1) Staphylococcus xylosus (KX946185.1)* | *S. xylosus (95%)* |
| ***S257*** | - | *Staphylococcus epidermidis (MT071633.1)* | *S. epidermidis (98%)* |
| ***S258*** | - | *Staphylococcus epidermidis (MT071633.1)* | *S. epidermidis (99%)* |
| ***S274*** | - | *Staphylococcus edaphicus (MN448412.1) Staphylococcus saprophyticus (MH144286.1)* | *S. saprophyticus (99%)* |
| ***S281*** | - | *Staphylococcus arlettae (MN851083.1) Staphylococcus saprophyticus subsp. bovis (LC511702.1)* | *S. saprophyticus (98%)* |
| ***S286*** | + | *Staphylococcus agnetis (MN314593.1) Staphylococcus hyicus (MF678941.1)* | *S. hyicus (93%)* |
| ***S296*** | - | *Staphylococcus cohnii (MT072172.1) Staphylococcus arlettae (MN851077.1)* | *S. cohnii (97%)* |
| ***S302*** | - | *Staphylococcus agnetis (MN314593.1) Staphylococcus hyicus (MF678941.1)* | *S. hyicus (99%)* |
| ***S317*** | - | *Staphylococcus agnetis (MN314593.1) Staphylococcus hyicus (MF678885.1)* | *S. hyicus (97%)* |
| ***S318*** | - | *Staphylococcus agnetis (MF678917.1) Staphylococcus hyicus (MF678885.1)* | *S. hyicus*  *(89%)* |
| ***S336*** | - | *Staphylococcus chromogenes (MT072191.1)* | *S. vitulinus*  *(91%)* |
| ***S344*** | - | *Staphylococcus agnetis (MK015776.1) Staphylococcus hyicus (MF678941.1)* | *S. hyicus (88%)* |
| ***S143*** | - | *Mammaliicoccus sciuri (MT072194.1)* | *S. sciuri (95%)* |
| ***S263*** | - | *Mammaliicoccus sciuri (KT260763.1)* | *S: sciuri (99%)* |
| ***S270*** | - | *Mammaliicoccus sciuri (MT072194.1)* | *S. sciuri (99%)* |

*GenBank accession numbers

**% probability of the identification
