# Supplementary material for: Genus-targeted markers for the taxonomic identification and monitoring of coagulase-positive and coagulase-negative Staphylococcus species
Source: World J Microbiol Biotechnol. 2024 Oct 3;40(11):333. doi: 10.1007/s11274-024-04121-9 (PMC11447098; doi:10.1007/s11274-024-04121-9)
Supplement: Supplementary file 2 — Supplementary Material 2 [file 11274_2024_4121_MOESM2_ESM.docx]

**S_2_**. Summary information of target genes used in this research

| **Gene** | **InterPro domains** | **GO terms** | **Description/Function** | **Phyletic profile** | **Evolutive rate** |  |
| --- | --- | --- | --- | --- | --- | --- |
| ***pta*** | Phosphate acetyltransferase | GO:0008959 phosphate acetyltransferase activity  GO:0006085 acetyl-CoA biosynthetic process | This protein is involved in step 2 of the subpathway that synthesizes acetyl-CoA from acetate. This subpathway is part of the pathway acetyl-CoA biosynthesis, which is itself part of Metabolic intermediate biosynthesis. | Present and single-copy in 43 species | 0.99 | |
| ***tpi*** | Triosephosphate isomerase | GO:0004807 triose-phosphate isomerase activity  GO:0006094 gluconeogenesis.  GO:0006096 glycolytic process | This protein is involved in the pathway gluconeogenesis, which is part of Carbohydrate biosynthesis. This protein is involved in step 1 of the subpathway that synthesizes D-glyceraldehyde 3-phosphate from glycerone phosphate | Present and single-copy in 43 species | 0.87 | |
| ***groES*** | groES chaperonin family (IPR020818) | GO:0006457 protein folding GO:0005737 cytoplasm | The chaperonins are 'helper' molecules required for correct folding and subsequent assembly of some proteins. These are required for normal cell growth, and are stress-induced, acting to stabilise or protect disassembled polypeptides under heat-shock conditions. | Present and single-copy in 49 species | 0.89 | |
| ***sarA*** | Transcriptional regulator sarA/Rot (IPR010166) | GO:0006355 regulation of transcription, DNA-templated | A global regulator with both positive and negative effects that controls expression of several virulence factors and biofilm formation process in a cell density-dependent manner | Present and single-copy in 49 species | 0.86 | |
| ***tufA*** | Translation elongation factor EFTu/EF1A, bacterial/organelle (IPR004541) | GO:0006414 translational elongation.  GO:0005525 GTP binding.  GO:0003746 translation elongation factor activity.  GO:0005622 intracellular | EF1A (or EF-Tu) is responsible for the selection and binding of the cognate aminoacyl-tRNA to the A-site (acceptor site) of the ribosome. Elongation factors are responsible for achieving accuracy of translation and EF1A is remarkably conserved throughout evolution. | Present and single-copy in 49 species | 0.77 | |
| ***gmK*** | Guanylate kinase | GO:0006163 purine nucleotide metabolic process.  GO:0004385 guanylate kinase activity | Guanylate kinase, also called GMP kinase, is essential for recycling GMP and indirectly, cGMP. This enzyme transfers a phosphate from ATP to GMP, yielding ADP and GDP. Guanylate kinase is a highly conserved monomer and is important for the activation of various antiviral drugs. | single copy in 43 species, multi-copy in 0 species | 0,78 | |
| ***glpF*** | Glycerol kinase | GO:0015267 channel activity-  GO:0016020 membrane | Enables the energy-independent facilitated diffusion, mediated by passage of a solute through a transmembrane aqueous pore or channel | single copy in 41 species, multi-copy in 0 species | 0,82 | |
| ***yqil*** | Acetyl coenzyme A acetyltransferase | GO:0003985 Catalysis of the reaction: 2 acetyl-CoA = CoA + acetoacetyl-CoA.  GO:0008152 The chemical reactions and pathways resulting in the formation of acetyl-CoA, a derivative of coenzyme A in which the sulfhydryl group is acetylated. | The chemical reactions and pathways, including anabolism and catabolism, by which living organisms transform chemical substances. Metabolic processes typically transform small molecules, but also include macromolecular processes such as DNA repair and replication, and protein synthesis and degradation. | single copy in 43 species, multi-copy in 0 species | 0,99 | |
