# Supplementary material for: Genus-targeted markers for the taxonomic identification and monitoring of coagulase-positive and coagulase-negative Staphylococcus species
Source: World J Microbiol Biotechnol. 2024 Oct 3;40(11):333. doi: 10.1007/s11274-024-04121-9 (PMC11447098; doi:10.1007/s11274-024-04121-9)
Supplement: Supplementary file 3 — Supplementary Material 3 [file 11274_2024_4121_MOESM3_ESM.docx]

**S_3_**. Staphylococcus species genomes used in the study

| **Specie** | **Strain** | **Coagulase response** | **Isolation source** | **NCBI Reference Sequence: genome feature** | **Size (Mb)** | **No. Protein** |
| --- | --- | --- | --- | --- | --- | --- |
| *S. xylosus* | HKUOPL8 | - | panda feces | NZ_CP007208.1 | 2.83 | 2258 |
| *S. warneri* | L37603 | - | Human skin | NZ_ACPZ00000000.1 | 2.42 | 2245 |
| *S. succinus* | DSM 14617 | - | plant and soil | NZ_LCSH00000000.1 | 2.88 | 2644 |
| *S. simulans* | CJ16 | - | Surgical Site Infection | NZ_LJSL00000000.1 | 2.67 | 2491 |
| *S. simiae* | CCM 7213 | - | Monkeys feces | NZ_AEUN00000000 | 2.58 | 2279 |
| *S. coagulans* | 1360-13 | + | Skin lesion of a dog | NZ_CP009470.1 | 2.43 | 2152 |
| *S. saprophyticus* | ATCC 15305 | - | ATCC 15305 | NC_007350.1 | 2.51 | 2351 |
| *S. pseudintermedius* | HKU10-03 | + | Skin lesion of a dog | NC_014925.1 | 2.61 | 2384 |
| *S. pasteuri* | SP1 | - | ID* | NC_022737 | 2.55 | 2381 |
| *S. microti* | DSM 22147 | - | Adult male liver | NZ_JXWY00000000.1 | 2.38 | 2143 |
| *S. lutrae* | ATCC 700373 | + | ATCC 700373 | NZ_CP020773.1 | 2.53 | 2204 |
| *S. lugdunensis* | HKU09-01 | - | Human skin | NC_013893.1 | 2.65 | 2425 |
| *S. intermedius* | NCTC 11048 | + | Nares of a pigeon | NZ_CAIB00000000 | 2.73 | 2530 |
| *S. hyicus* | ATCC 11249 | +/- | Exudative Epidermitis in Swine | NZ_CP008747.1 | 2.47 | 2262 |
| *S. hominis* | SK119 | - | Human skin | NZ_ACLP00000000.1 | 2.22 | 2106 |
| *S. haemolyticus* | JCSC1435 | - | pharyngeal smear of a human | NC_007168.1 | 2.68 | 2484 |
| *S. gallinarum* | DSM 20610 | - | Chicken skin | NZ_JXCF00000000 | 3.1 | 2864 |
| *S. equorum* | Mu2 | - | french smear-ripened cheese | NZ_CAJL00000000 | 2.9 | 2741 |
| *S. epidermidis* | ATCC 12228 | - | ATCC 12228 | NC_004461.1 | 2.4 | 2482 |
| *S. delphini* | 8086 | + | Trachea of a horse | NZ_CAIA00000000.1 | 2.5 | 2315 |
| *S. cohnii* | hu-01 | - | Human skin | NZ_AYOS00000000.2 | 1.6 | 2476 |
| *S. chromogenes* | MU 970 | - | Cow's rear mammary | NZ_JMJF00000000.1 | 5.5 | 2175 |
| *S. carnosus subsp. Carnosus* | TM300 | - | Meat culture | NC_012121.1 | 2.5 | 2359 |
| *S. caprae* | M23864:W1 | - | Human Microbiome | ACJB00000000.1 | 2.6 | 2398 |
| *S. capitis* | SK14 | - | Human skin | NZ_ACFR00000000.1 | 2.4 | 2272 |
| *S. auricularis* | DSM 20609 | - | Human external ear | NZ_LLER00000000.1 | 2.2 | 1970 |
| *S. aureus* | RF122 | + | Cow with mastitis | NC_007622.1 | 2.7 | 2591 |
| *S. arlettae* | CVD059 | - | Human blood | NZ_ALWK00000000.1 | 2.56 | 2308 |
| *S. argenteus* | MSHR1132 | - | blood culture of an indigenous woman | NC_016941 | 2.76 | 2568 |
| *S. agnetis* | CBMRN20813338 | +/- | Cow with mastitis | PPQF00000000 | 3.43 | 2267 |
| *Mammaliicoccus vitulinus* | F1028 | - | Fermented soybean | NZ_AJTR00000000.1 | 2.55 | 2459 |
| *Mammaliicoccus sciuri* | DSM 20345 | - | Skin of squirrel | NZ_LEOS00000000.1 | 2.78 | 2666 |
| *Mammaliicoccus lentus* | MF1862 | - | Poultry | NZ_LSKY00000000.1 | 2.01 | 2490 |

ID: Indeterminate*
