# Supplementary material for: Genus-targeted markers for the taxonomic identification and monitoring of coagulase-positive and coagulase-negative Staphylococcus species
Source: World J Microbiol Biotechnol. 2024 Oct 3;40(11):333. doi: 10.1007/s11274-024-04121-9 (PMC11447098; doi:10.1007/s11274-024-04121-9)
Supplement: Supplementary file 4 — Supplementary Material 4 [file 11274_2024_4121_MOESM4_ESM.docx]

**S_4_.** Taxonomic identification of the isolates based on house-keeping genes using Blast.

| **Gene** | **Isolate** | **bp** | **Organism** | **Max Score** | **Total Score** | **Query Cover** | **E value** | **Percent Identity** |
| --- | --- | --- | --- | --- | --- | --- | --- | --- |
| ***pta*** | *S004* | 325 | [*S. devriesei*](https://www.ncbi.nlm.nih.gov/Taxonomy/Browser/wwwtax.cgi?id=586733)  strain GYZ2  *CP130489.1* | 595 | 595 | 99% | 2e-165 | 100.00% |
|  | *S007* | 325 | *S. haemolyticus* strain ATCC 29970 (CP035291.1) | 590 | 590 | 100% | 2,00E+164 | 99,38% |
|  | *S016* | 334 | *S. chromogenes* strain 20B (CP031471.1) | 608 | 608 | 99% | 6,00E+170 | 99,70% |
|  | *S060* | 350 | *S. chromogenes* strain 20B (CP031471.1) | 625 | 625 | 100% | 6,00E+175 | 98,57% |
|  | *S081* | 331 | S. warneri strain FDAARGOS (CP054017) | 597 | 597 | 100% | 1,00E+166 | 99.09% |
|  | *S143* | 326 | *Mammaliicoccus sciuri,* FDAARGOS_285 (CP022046.2) | 603 | 603 | 100% | 3,00E-168 | 100% |
|  | *S196* | 329 | *S. xylosus* strain 2 (CP031275.1) | 564 | 564 | 100% | 1,00E-156 | 97,57% |
|  | *S257* | 326 | *S. epidermidis* strain ATCC 12228 (CP043845.1) | 601 | 601 | 99% | 9,00E-168 | 100% |
|  | *S258* | 343 | *S. epidermidis* strain ATCC 12228 (CP043845.1) | 610 | 610 | 99% | 2,00E-170 | 98,82% |
|  | *S263* | 323 | *Mammaliicoccus sciuri,* strain FDAARGOS_285 (CP022046.2) | 597 | 597 | 100% | 1,00E-166 | 100% |
|  | *S270* | 328 | *Mammaliicoccus sciuri* strain B9-58B (CP041879.1) | 595 | 595 | 100% | 4,00E-166 | 99,39% |
|  | *S274* | 326 | *S. saprophyticus* strain IUHSS04 (KM454817.1) | 603 | 603 | 100% | 3,00E-168 | 100% |
|  | *S281* | 325 | *S. saprophyticus* strain 1A (CP031196.1) | 601 | 601 | 100% | 9,00E-168 | 100% |
|  | *S286* | 325 | *S. agnetis* strain 12B (CP031266.1) | 579 | 579 | 99% | 4,00E-161 | 99,07% |
|  | *S296* | 323 | *Staphylococcus cohnii strain* SNUDS-2  (CP019597.1) | 564 | 564 | 100% | 7e-156 | 95.98% |
|  | *S302* | 341 | *S. agnetis* strain 12B (CP031266.1) | 601 | 601 | 99% | 1,00E-167 | 98,59% |
|  | *S317* | 328 | *S. agnetis* strain 12B (CP031266.1) | 584 | 584 | 100% | 1,00E-162 | 98,78% |
|  | *S318* | 324 | *S. agnetis* strain 12B (CP031266.1) | 582 | 582 | 99% | 3,00E-162 | 99,38% |
|  | *S336* | 326 | *S. chromogenes* strain 34B (CP031470.1) | 586 | 586 | 100% | 3,00E-163 | 99,08% |
|  | *S344* | 326 | *S. agnetis* strain 12B (CP031266.1) | 580 | 580 | 99% | 1,00E-161 | 99,07% |
|  | ATCC25923 | 326 | *S. aureus* strain UP_338 (CP047851.1) | 546 | 546 | 94% | 4.4e151 | 99% |
| ***groEs*** | *S004* | 172 | [*S. devriesei*](https://www.ncbi.nlm.nih.gov/Taxonomy/Browser/wwwtax.cgi?id=586733)  strain GYZ2  CP130489.1 | 261 | 261 | 100% | 3e-65 | 98.63% |
|  | *S007* | 156 | *S. haemolyticus* strain VB5326 (CP045137.2) | 244 | 244 | 93% | 8,00E-61 | 96,58% |
|  | *S016* | 158 | *S. chromogenes* strain 17A (CP031274.1) | 276 | 276 | 100% | 3,00E-70 | 98,10% |
|  | *S060* | 164 | *S. chromogenes* strain 17A (CP031274.1) | 287 | 287 | 98% | 1,00E-73 | 98,76% |
|  | *S081* | 158 | *S. warneri strain* FDAARGOS (CP054017.1) | 287 | 287 | 100% | 1,00E-73 | 99.37% |
|  | *S143* | 147 | *Mammaliicoccus sciuri* strain FDAARGOS_285 (CP022046.2) | 261 | 261 | 97% | 3e-65 | 99.31% |
|  | *S196* | 164 | *S. saprophyticus* strain 1A (CP031196.1) | 250 | 250 | 100% | 2,00E-62 | 93,90% |
|  | *S257* | 168 | *S. epidermidis* strain ATCC 12228 (CP043845.1) | 292 | 292 | 99% | 3,00E-75 | 98,20% |
|  | *S258* | 172 | *S. epidermidis* strain ATCC 12228 (CP043845.1) | 294 | 294 | 99% | 8,00E-76 | 97,66% |
|  | *S263* | 168 | *Mammaliicoccus sciuri* strain FDAARGOS_285 (CP022046.2) | 278 | 278 | 99% | 8,00E-71 | 96,41% |
|  | *S270* | 161 | [*Mammaliicoccus sciuri strain SSC-7107 (CP071138.1)*](https://www.ncbi.nlm.nih.gov/nucleotide/CP071138.1?report=genbank&log$=nucltop&blast_rank=3&RID=9XMGZU3P01N) | 265 | 265 | 98% | 8,00E-67 | 96.84%% |
|  | *S274* | 173 | *S. saprophyticus* strain 1A (CP031196.1) | 296 | 296 | 98% | 2,00E-76 | 97,66% |
|  | *S281* | 172 | *S. saprophyticus* strain 1A (CP031196.1) | 296 | 296 | 99% | 2,00E-76 | 97,66% |
|  | *S286* | 169 | *S. agnetis* strain 12B (CP031266.1) | 265 | 265 | 99% | 7,00E-67 | 94,64% |
|  | *S296* | 173 | *S. cohnii* strain FDAARGOS_334 (CP027422.1) | 285 | 285 | 98% | 5,00E-73 | 96,49% |
|  | *S302* | 173 | *S. agnetis* strain 12B (CP031266.1) | 274 | 274 | 98% | 1,00E-69 | 95,32% |
|  | *S317* | 157 | *S. agnetis* strain 12B (CP031266.1) | 281 | 281 | 100% | 6,00E-72 | 98,73% |
|  | *S318* | 149 | *S. agnetis* strain 12B (CP031266.1) | 244 | 244 | 100% | 7,00E-61 | 95,97% |
|  | *S336* | 148 | *S. chromogenes* strain 34B (CP031470.1) | 252 | 252 | 100% | 4,00E-63 | 97,30% |
|  | *S344* | 143 | *S. agnetis* strain 12B (CP031266.1) | 243 | 243 | 100% | 2,00E-60 | 97,20% |
|  | ATCC25923 | 168 | *S. aureus* strain UP_338 (CP047851.1) | 233 | 233 | 99% | 1,00E-57 | 99% |
| ***tuf*** | *S004* | 395 | *S. devriesei* strain CCUG 58238 (MF678988.1) | 721 | 721 | 100% | 0.0 | 99,49% |
|  | *S007* | 395 | *S. haemolyticus* strain VB5326 (CP045137.2) | 725 | 725 | 100% | 0.0 | 99,75% |
|  | *S016* | 396 | *S. chromogenes* strain PCM 2193 (MF678984.1) | 732 | 732 | 100% | 0.0 | 100% |
|  | *S060* | 403 | *S. chromogenes* strain 34B (CP031470.1) | 745 | 745 | 100% | 0.0 | 100% |
|  | *S081* | 393 | *S. warneri* strain FDAARGOS (CP054017) | 706 | 706 | 100% | 0.0 | 98.98% |
|  | *S143* | 377 | *Mammaliicoccus sciuri* strain B9-58B (CP041879.1) | 697 | 697 | 100% | 0.0 | 100% |
|  | *S196* | 308 | *S. saprophyticus* strain KW45 (MF620945.1) | 558 | 558 | 98% | 5,00E-155 | 100% |
|  | *S257* | 383 | *S. epidermidis* strain ATCC 12228 (CP043845.1) | 704 | 704 | 100% | 0.0 | 99,74% |
|  | *S258* | 385 | *S. epidermidis* strain ATCC 12228 (CP043845.1) | 712 | 712 | 100% | 0.0 | 100% |
|  | *S263* | 399 | *Mammaliicoccus sciuri* strain B9-58B (CP041879.1) | 737 | 737 | 100% | 0.0 | 100% |
|  | *S270* | 399 | *Mammaliicoccus sciuri* strain B9-58B (CP041879.1) | 737 | 737 | 100% | 0.0 | 100% |
|  | *S274* | 403 | *S. saprophyticus* strain 1A (CP031196.1) | 745 | 745 | 100% | 0.0 | 100% |
|  | *S281* | 373 | *S. saprophyticus* strain 1A (CP031196.1) | 680 | 680 | 99% | 0.0 | 99,73% |
|  | *S286* | 398 | *S. agnetis* strain 12B (CP031266.1) | 736 | 736 | 100% | 0.0 | 100% |
|  | *S296* | 386 | *S. cohnii* strain FDAARGOS_334 (CP027422.1) | 713 | 713 | 100% | 0.0 | 100% |
|  | *S302* | 398 | *S. agnetis* strain 12B (CP031266.1) | 736 | 736 | 100% | 0.0 | 100% |
|  | *S317* | 380 | *S. agnetis* strain 12B (CP031266.1) | 697 | 697 | 100% | 0.0 | 99,74% |
|  | *S318* | 341 | *S. agnetis* strain 12B (CP031266.1) | 630 | 630 | 100% | 1,00E-176 | 100% |
|  | *S336* | 380 | *S. chromogenes* strain 34B (CP031470.1) | 695 | 695 | 100% | 0.0 | 99,74% |
|  | *S344* | 381 | *S. agnetis* strain 12B (CP031266.1) | 704 | 704 | 100% | 0.0 | 100% |
|  | ATCC25923 | 400 | *S. aureus* strain SR153 (CP048643.1) | 651 | 651 | 93% | 0.0 | 100% |
| ***tpi*** | *S004* |  | [*S. devriesei*](https://www.ncbi.nlm.nih.gov/Taxonomy/Browser/wwwtax.cgi?id=586733)  strain GYZ2  CP130489.1 | 708 | 708 | 99% | 0.0 | 99.74% |
|  | *S007* | 338 | *S. haemolyticus* strain VB19458 (CP045187.2) | 614 | 614 | 100% | 1,00E-171 | 99,41% |
|  | *S016* | 384 | *S. chromogenes* strain 20B (CP031471.1) | 704 | 704 | 100% | 0.0 | 99,74% |
|  | *S060* | 384 | *S. chromogenes* strain 20B (CP031471.1) | 704 | 704 | 100% | 0.0 | 99.74% |
|  | *S081* | 324 | *S. warneri* strain FDAARGOS (CP054017.1) | 593 | 593 | 100% | 2,00E-165 | 99.69% |
|  | *S143* | 386 | *Mammaliicoccus sciuri* strain B9-58B (CP041879.1) | 713 | 713 | 100% | 0.0 | 100% |
|  | *S196* | 383 | *S. xylosus* strain 2 (CP031275.1) | 601 | 601 | 98% | 1,00E-167 | 95,48% |
|  | *S257* | 381 | *S. epidermidis* strain ATCC 12228 (CP043845.1) | 704 | 704 | 100% | 0.0 | 100% |
|  | *S258* | 385 | *S. epidermidis* strain ATCC 12228 (CP043845.1) | 712 | 712 | 100% | 0.0 | 100% |
|  | *S263* | 384 | *Mammaliicoccus sciuri* strain B9-58B (CP041879.1) | 704 | 704 | 100% | 0.0 | 99,74% |
|  | *S270* | 384 | *Mammaliicoccus sciuri* strain GDK8D55P (CP065960.1) | 710 | 710 | 100% | 0.0 | 100% |
|  | *S274* | 381 | *S. saprophyticus* isolate 883 (LT963436.1) | 704 | 704 | 100% | 0.0 | 100% |
|  | *S281* | 384 | *S. saprophyticus* strain ATCC 15305 (CP035294.1) | 710 | 710 | 100% | 0.0 | 100% |
|  | *S286* | 382 | *S. agnetis* strain 12B (CP031266.1) | 678 | 678 | 100% | 0.0 | 98,69% |
|  | *S296* | 384 | *S. cohnii* strain FDAARGOS_334 (CP027422.1) | 688 | 688 | 100% | 0.0 | 98,96% |
|  | *S302* | 381 | *S. agnetis* strain 12B (CP031266.1) | 688 | 688 | 100% | 0.0 | 99,21% |
|  | *S317* | 384 | *S. agnetis* strain 12B (CP031266.1) | 682 | 682 | 100% | 0.0 | 98,70% |
|  | *S318* | 384 | *S. agnetis* strain 12B (CP031266.1) | 682 | 682 | 100% | 0.0 | 98,70% |
|  | *S336* | 336 | *S. chromogenes* strain 34B (CP031470.1) | 593 | 593 | 100% | 2,00E-165 | 98,51% |
|  | *S344* | 385 | *S. agnetis* strain 12B (CP031266.1) | 684 | 684 | 100% | 0.0 | 98,70% |
|  | *ATCC 252923* | 383 | *S. aureus* strain UP_338 (CP047851.1) | 440 | 440 | 95% | 3,00E-119 | 100% |
| ***sarA*** | *S004* | 138 | *S. hominis* strain 19A (CP031277.1) | 187 | 187 | 97% | 1,00E-43 | 91,79% |
|  | *S007* | 153 | *S. haemolyticus* strain VB5326 (CP045137.2) | 239 | 239 | 100% | 3,00E-59 | 94,12% |
|  | *S016* | 154 | *S. chromogenes* strain 17A (CP031274.1) | 267 | 267 | 99% | 2,00E-67 | 97,39% |
|  | *S060* | 138 | *S. chromogenes* strain 17A (CP031274.1) | 250 | 250 | 100% | 1,00E-62 | 99,28% |
|  | *S081* | 152 | *S. warneri* strain FDAARGOS (CP054017.1) | 255 | 255 | 94% | 3,00E-64 | 98,61% |
|  | *S196* | 162 | *S. saprophyticus* strain ATCC 15305 (CP035294.1) | 237 | 237 | 100% | 1,00E-58 | 92,59% |
|  | *S257* | 160 | *S. epidermidis* strain ATCC 12228 (CP043845.1) | 272 | 272 | 100% | 4,00E-69 | 96,88% |
|  | *S258* | 162 | *S. epidermidis* strain ATCC 12228 (CP043845.1) | 267 | 267 | 100% | 2,00E-67 | 95,68%% |
|  | *S274* | 137 | *S. saprophyticus* strain ATCC 15305 (CP035294.1) | 244 | 244 | 100% | 6,00E-61 | 98,54% |
|  | *S281* | 156 | *S. saprophyticus* strain ATCC 15305 (CP035294.1) | 268 | 268 | 100% | 5,00E-68 | 97,44% |
|  | *S286* | 156 | *S. agnetis* strain 12B (CP031266.1) | 263 | 263 | 99% | 2,00E-66 | 96,77% |
|  | *S296* | 152 | *S. cohnii* strain FDAARGOS_334 (CP027422.1) | 257 | 257 | 100% | 1,00E-64 | 96,71% |
|  | *S302* | 138 | *S. agnetis* strain 12B (CP031266.1) | 255 | 255 | 100% | 3,00E-64 | 100% |
|  | *S317* | 153 | *S. agnetis* strain 12B (CP031266.1) | 255 | 255 | 99% | 3,00E-64 | 96,71% |
|  | *S318* | 154 | *S. agnetis* strain 12B (CP031266.1) | 261 | 261 | 98% | 8,00E-66 | 97,37% |
|  | *S336* | 157 | *S. chromogenes* strain 34B (CP031470.1) | 265 | 265 | 99% | 6,00E-67 | 96,79% |
|  | *S344* | 153 | *S. agnetis* strain 12B (CP031266.1) | 259 | 259 | 96% | 3,00E-65 | 97,97% |
|  | ATCC25923 | 159 | *S. aureus* Tokyo12482 (AP019713.1) | 230 | 230 | 95% | 2,00E-56 | 98,01% |
