# Supplementary material for: Genus-targeted markers for the taxonomic identification and monitoring of coagulase-positive and coagulase-negative Staphylococcus species
Source: World J Microbiol Biotechnol. 2024 Oct 3;40(11):333. doi: 10.1007/s11274-024-04121-9 (PMC11447098; doi:10.1007/s11274-024-04121-9)

**S6**.

Supplementary file 6. Amplification *sarA* gene in Staphylococcus strains S196, S281 and S286, amplified using Illumina MiSeq technology.


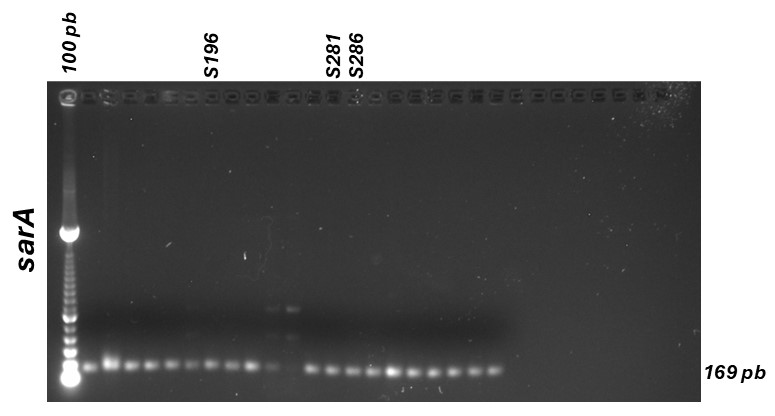

Supplement: Supplementary file 6 — Supplementary Material 6 [file 11274_2024_4121_MOESM6_ESM.docx]
